# Decision-making and performance in the Iowa Gambling Task: recent ERP findings and clinical implications

**DOI:** 10.3389/fpsyg.2025.1492471

**Published:** 2025-03-19

**Authors:** Ashley Latibeaudiere, Santo Butler, Max Owens

**Affiliations:** TROPICS Lab, Department of Psychology, University of South Florida, St. Petersburg, FL, United States

**Keywords:** IGT, EEG, ERP, Iowa Gambling Task, clinical

## Abstract

The Iowa Gambling Task (IGT) is a widely used tool for assessing decision-making in clinical populations. In each trial of the task, participants freely select from different playing card decks that vary in the magnitude and frequency of rewards and punishments. Good decks offer relatively smaller rewards on each trial yielding greater overall winnings while bad decks result in a net loss over time as high penalties negate any rewards earned. Comparing participants’ rate of selecting good to bad decks can provide insight into learning in uncertain conditions across time. However, inconsistent patterns of deficits and learning within clinical and control populations are often observed in the task (eg., in depression). Thus, a clearer mechanistic understanding of the IGT is needed to fully understand the decision-making process. The goal of the current review is to synthesize the predominant empirical and theoretical literature of the IGT using event-related potentials (ERPs) derived from electroencephalogram (EEG). The review then explores how modifications of the IGT allow for event-related potentials to be captured at each stage of decision-making. Lastly, the review discusses how the modified version with ERPs can be further applied to directly assess the impact of emotion processing on decision-making, using findings from depression research as an example.

## 1 Introduction

The Iowa Gambling Task (IGT) is a popular, clinically validated tool to assess the nature of the human decision-making process (Steingroever et al., 2013). In the IGT, participants attempt to maximize their earnings on a loan of money (Bechara et al., 1994). They are presented with four decks, of which two are advantageous. Participants are told that some cards are more beneficial than others. With each selection, participants win or lose a set amount of money. Advantageous decks (C and D) begin with small gains but end with even smaller losses, resulting in a net gain over time. The disadvantageous decks (A and B) result in a net loss as large gains are negated by larger future losses. The frequency of losses and gains also vary across decks as Decks B and D result in less frequent but high-magnitude losses, while decks A and C result in smaller but more frequent losses (see Table 1). Typically, participants show learning in the task by decreasing selections of disadvantageous decks and increasing the selection of advantageous decks across several blocks of trials (Bechara et al., 1994; Dong et al., 2016). As such, it is argued that the task relies on constructing long-term probabilistic associations of four options across time, a process that has been proposed to mimic real-life decision-making (Bechara et al., 2000a).

**Table 1:** Table showing the characteristics of each deck in the standard IGT.

	A	B	C	D
% Wins	50%	90%	50%	90%
Magnitude of wins	100	100	50	50
Magnitude of losses	−150 to −350	−1,250	−25 to −75	−250
Expected profit for 10 cards	−250	−250	250	250

Adapted from Bechara et al. (1994).

There are several behavioral measures often used to characterize performance on the IGT. Generally, performance is calculated based on the difference between the number of choices from advantageous(C, D) and disadvantageous decks (A, B; Bechara et al., 1994; Ernst et al., 2002). Positive scores indicate good performance as individuals make a greater selection of choices from advantageous decks while negative scores indicate poor performance because of a greater reliance on disadvantageous decks. Typically, the change in net score across blocks of 20 trials is computed, where an increase in net score is interpreted to reflect learning in the task (Bechara et al., 2000b; Bowman et al., 2005; DeDonno and Demaree, 2008).

Since its introduction, the IGT has been widely used to characterize decision making in clinical populations. Bechara et al. (2000b) found that while the control group increased their net score by selecting more advantageous decks across time, those with ventromedial prefrontal cortex (vmPFC) damage continued in selecting from disadvantageous decks, resulting in a negative net score. Research has shown that several other clinical groups have difficulty learning on the IGT, including those with amygdala damage (Bechara et al., 1999), substance abuse disorder (Balconi et al., 2014), gambling disorder (Kovács et al., 2017), schizophrenia (Shurman et al., 2005), obsessive compulsive disorder (Cavallaro et al., 2003), and others (for a full review see Buelow and Suhr, 2009).

### 1.1 Factors underlying IGT performance—the somatic marker and cognitive hypotheses

#### 1.1.1 The somatic marker hypothesis

Learning on the IGT has been argued to be driven in part by emotion or hunches, developed through prior exposure to rewards and losses (Bechara et al., 2000a). In this view, repeated exposure to stimuli elicits somatic signals tied to emotional states, which in turn biases one’s decision. This theory, known as the somatic marker hypothesis, was developed based on the physiological responses observed in those with vmPFC damage while playing the IGT (Bechara and Damasio, 2005). Using skin conductance responses(SCR), Bechara et al. (1999) showed that those with vmPFC damage responded to instances of wins and losses, but failed to develop anticipatory SCR necessary to distinguish between good and bad decks. The vmPFC is thought to be critical in integrating associations from different brain regions including the amygdala to generate somatic markers (Bechara et al., 1999; Bechara and Damasio, 2005). Therefore, damage to this region is argued to result in an inability to generate the appropriate skin conductance response necessary to guide the avoidance of bad decks. Importantly, the somatic marker hypothesis does not infer that cognition has no role in decision-making but that emotion precedes and therefore contributes to the development of explicit knowledge (Buelow and Suhr, 2009).

#### 1.1.2 Cognitive hypothesis

A key tenet of the somatic marker hypothesis is that learning on the IGT relies on the development of somatic markers (Bechara et al., 1997). However, when sampling verbal reports during game play it has been found that generally participants have conscious knowledge of the deck outcomes that is not entirely reflected in their behavior (Maia and McClelland, 2004). Therefore, the authors proposed that conscious knowledge and behavior may result from partially separate mechanisms (Maia and McClelland, 2004). Although, it may be expected that conscious knowledge, somatic markers and behavior interact. In this regard, there is evidence that high and low conceptual knowledge of the deck outcomes are associated with different patterns of neural reactivity in the task using ERPs (Marino and Mantini, 2024; Dong et al., 2016). As a result, cognition might have a greater role guiding behavior on the IGT than initially thought (Table 2).

**Table 2:** Table showing the event-related potentials (ERPs) investigated in studies using the modified Iowa Gambling Task (IGT) and standard IGT.

Task	Author	Population and factor investigated	Participant characteristics	Stage	ERP	Major finding
Modified IGT	Guo et al. (2019)	Control group, Social distance	*n* = 57 (right-handed; 36 female); M = 20.263, SD = 1.987.	Choice evaluation Response Feedback	P300 DPN FRN P300	Disadvantageous (Disadv.) > Advantageous (Adv.) decks Adv: High social dist > low social dist. Disadv: Low social > high social dist. Disadv.: High social distance > low social dist. Playing for oneself > stranger Win > Loss Playing for oneself > stranger
	Dong et al. (2016)	Control group, Cognitive flexibility (cf)	*n* = 34 (right-handed; 22 female); M = 21.62, SD = 2.5; range = 19–26	Choice evaluation Response Feedback	P300 DPN FRN	High cf. group: Disadv deck: Parietal & central > frontal Adv deck: parietal > central > frontal. Low cf. group: no diff between decks & regions. High cf. group: Greater -ve peak/higher DPN for passing > playing Low cf. group: Higher DPN for playing > passing High cf. group: Loss > Win Low cf. group: Win > Loss
	Cui et al. (2013)	Control group, Disadvantageous vs. advantageous decks	*n* = 26 (14 male); M = 22.35, SD = 1.74; range = 19 to 25	Choice Evaluation Response selection Feedback	P300 DPN FRN P300	Adv: P300 in left > P300 in right hemisphere. Disadv: P300 in left < P300 right hemisphere. Higher DPN for pass > play, specifically for disadvantageous decks Loss > Win Loss > Win
Standard IGT	Zhang et al. (2024)	Mild cognitive Impairment (MCI)	*n* = 49 MCI = 24, Control = 25	Feedback	FRN P300	Win > Loss Smaller FRN in MCI < Control Smaller P300 in MCI < Control
	Azcárraga-Guirola et al. (2017)	Multiple Sclerosis (MS)	*n* = 25; MS = 16 (M = 39.4, SD = 12.6, 6 male) Control =19 (M = 38.3, SD = 10.3, 6 male)	Response selection Feedback	P300 FRN P300	no diff between Multiple sclerosis (MS) and control groups Control: Nothing > Loss > Win MS: no diff. Control: Loss < Nothing MS: no diff.
	Balconi et al. (2015)	Control group, Behavioral Activating System (BAS)	*n* = 22 (10 female); M = 23.78, SD = 2.60; range = 19–25	Feedback	FRN P300	Loss > Win High Behavioral Activating System (BAS): no diff between loss & win Loss > Win High BAS: less of a diff between loss & win
	Mapelli et al. (2014)	Parkinson’s	*n* = 30 (11 M); Control = 15; (M = 60.7, SD = 9.8, range = 43–77) Parkinson’s = 15; (M = 61.4, SD = 9.6, range = 41–73)	Feedback	P200 FRN P300	Control: no diff win vs. loss Parkinson’s: no diff for win vs. loss Control: Win > Loss Parkinson’s: no diff for win vs. loss Control: Win > Loss Parkinson’s: no diff for win vs. loss
	Tamburin et al. (2014)	Chronic Back pain	*n* = 48; Control = 24 (14 female) (M = 46.1, SD = 17.5, range = 23–71) Chronic Back Pai*n* = 24 (9 female), (M = 47.7, SD = 9.1, range = 35–69)	Feedback	FRN P300	Control: n.s. Win > Loss Chronic back pain: Win < Loss Control: Win > Loss Chronic back pain: no diff between win & loss
	Schuermann et al. (2011)	Borderline Personality Disorder (BPD)	*n* = 36; Control = 18 (16 female); (M = 27.28, SD = 6.61); BPD = 18 (16 female), (M = 29.11, SD = 8.06)	Feedback	FRN P300	Control: Loss > Win BPD: no diff between loss & win; smaller FRN compared to control Control: no diff win vs. loss BPD: Loss > Win

This list is a sample of all the studies that ERP studies that have used the IGT. Please see Xu and Huang, 2020 and Chandrakumar et al., 2018 for a more complete list of publications.

Despite this support, there is less clarity on the exact cognitive processes underlying decision-making on the IGT. Some studies demonstrate that working memory, attention and cognitive flexibility (i.e., abstraction and set-shifting) predicts performance (Cui et al., 2015; Bechara and Martin, 2004; Tamburin et al., 2014; Brand et al., 2007; Lehto and Elorinne, 2003; Dong et al., 2016), while others find no impact of these processes on the IGT (Overman et al., 2004; Toplak et al., 2010). Typically, positive correlations between performance on the IGT and another task are used to draw inferences about its underlying cognitive processes. However, a drawback of this approach is that performance on these cognitive tasks might require a mixture of cognitive processes. For instance, although the Wisconsin Card Sorting task (WCST) has often been used as a measure of cognitive flexibility, it involves a complex interplay of other processes such as working memory, inhibition, and attention (Miles et al., 2021; Buchsbaum et al., 2005). To identify the specific processes that underlie individual behavior and learning in the task, some theorists have argued for a cognitive modelling approach (Busemeyer and Stout, 2002).

##### 1.1.2.1 Computational modelling of the IGT

Generally, computational modelling is the decomposition of trial-level choices into separate processes using theory grounded mathematical functions. For instance, according to the Expectancy Valence Learning Model (EVL), deck choices are tested for errors and updated across trials to align with an individual’s evolving valence expectations (Busemeyer and Stout, 2002). By tracking how an individual’s decision-making in the IGT responds to errors or success across the entire gameplay, the EVL attempts to explain underlying processes by fitting participants’ choices into the model parameters thought to reflect decision-making components of their cognition, such as: motivation, recency, and choice sensitivity (Busemeyer and Stout, 2002; Koritzky et al., 2014). In this view, motivation describes an individual’s sensitivity towards gains and losses in response to the feedback resulting from their choice. Recency captures the tendency to prioritize recent experience over its entire history. Lastly, choice sensitivity explains the consistency an individual has with their choices across trials, with low levels of choice sensitivity indicating random or exploratory behavior (Busemeyer and Stout, 2002). The greater these parameters “fit” the data, the more variance in IGT decision-making can be explained. This allows for differences evident in IGT play style to be directly partitioned and associated with relevant behavioral or emotional characteristics. For example, EVL research has shown that participants who had higher sensitivity to reward in their IGT choices had greater attrition rates in a weight management program. This result is thought to imply that these populations may have their eating tendencies increased by underlying reward driven processes, potentially making it more difficult to adhere to a treatment intervention (Koritzky et al., 2014).

However, modelling approaches that formalize choices across the entire gameplay may overly assume that advantageous decision-making in the IGT is a consequence of continuous learning of deck probabilities. Trial-level strategies, which are sensitive only to the outcome of the previous choice, may be a factor in the IGT (Cassotti et al., 2011; Worthy et al., 2013). The Win Play Lose Shift (WPLS) model captures participants’ tendency to play on decks which they had won in the most recent trial, but shift away once they have lost. When directly compared, this model provided a better relative fit to IGT data than the EVL model and performed similarly to another reinforcement model where the expectancies of all decks decay, or are discounted over time (PVL; Prospect Valence Learning model; Worthy et al., 2013). Furthermore, the onset and duration of these variations in play styles may be associated with their own individual differences. For example, research exploring strategic adjustment in the task by computing the frequency of response-switching following gains and losses found that adults tended to stay on the same deck after wins more frequently than children and adolescents. Conversely, children switched more after losses and gains relative to adults and only for losses relative to adolescents, who tended to display a less extreme tendency towards staying with wins and shifting with losses (Cassotti et al., 2011). At the end of the trial period, adults were observed to increase the number of advantageous deck selections across the task, whereas children and adolescents did not show this trend (Cassotti et al., 2011). Therefore, a large proportion of participant behavior can be explained by this relatively simple, more reflexive model that highlights strategic style rather than an elaborate cognitive assessment. More significantly, participants may be employing both strategic play styles and learned associations in the IGT. Relatively more flexible models have been developed to emulate this type of human performance. For instance, Iglesias et al. (2012) developed a knowledge-based model that integrates participants trial over trial experiences, overall deck contingencies, and how both may interact across gameplay. In this approach, it may be possible to model every possible decision outcome in a given task similar to the connectionist neural network approach of representing knowledge. Thus, in principle, models such as MAIDEN-IGT may offer the flexibility to capture changing playstyles while characterizing individual differences. However, neural networks risk overfitting data (Bejani and Ghatee, 2021), which can limit generalization of their outcomes to cognitive function.

Furthermore, outcome based performance indicators alone may not be enough to understand cognitive mechanisms at play in the IGT. Investigating process-based indicators might provide further insight into the patterns underlying individuals’ choices, which might be obscured when solely focusing on net score. Switching rates highlight differences in performance while serving as a more consistent measure of decision-making across tasks than deck choices (Yechiam, 2020). Zeif et al. (2023) found that people with autism spectrum disorder show a 13.5% increase in switching behavior but demonstrate a similar rate of disadvantageous card selections when compared to the control group. Their results suggested that this difference in behavior was more likely due to exploration than loss sensitivity or implicit learning deficits. An additional indicator to investigate individual differences is the rate of selection from decks differing in loss frequency, which might clarify how sensitivity to losses drives choice selection. Women are more likely to choose risky deck B over advantageous deck C because of a lower frequency of losses (Garon and Longard, 2015). In fact, a literature review by Steingroever et al. (2013) found that 13 of 17 studies indicated that participants chose risky deck B as much as advantageous deck D, indicating that loss frequency might be a more common factor influencing decision making than previously thought. As a result, switching and response to loss frequency uncovers additional individual differences trends, which is data that can improve upon current computational models. Computational approaches such as the EVL and PVL do not accurately predict these aspects of the participant’s behavior, underscoring the need for more comprehensive models (Steingroever et al., 2013). Interestingly, a model based on switching was found to be more accurate in predicting choices than the EVL and Bayesian models (Zhao and Costello, 2007), highlighting the potential benefit of integrating these metrics to further explore the nature of performance in the task. Moreover, more work is needed to determine how these processes relate to explicit awareness of the reward contingencies on the IGT (Zeif et al., 2023) and how well they map on to neural correlates in the brain.

### 1.2 Neurological basis of the IGT based on lesion and fMRI studies

Neuroimaging and brain lesion studies assessing performance demonstrate that multiple brain regions work in concert during the IGT, suggesting that multiple cognitive processes contribute to performance. These regions involve those associated with reward and emotional processing, and executive function (Li et al., 2010; see Lin et al., 2008 for a review). fMRI studies have shown enhanced activity in reward-processing areas such as the striatum of the basal ganglia, cerebellum, and thalamus (Lawrence et al., 2009). Areas governing emotional responses to stimuli, such as the amygdala, are also active (Li et al., 2010; Ernst and Paulus, 2005; Lawrence et al., 2009; Lin et al., 2008). Critically, lesions to the amygdala result in no SCRs to either wins or losses, resulting in poor performance (Bechara et al., 1999). Meanwhile, damage to the vmPFC, which is involved in emotional processing, results in an inability to generate SCRs to anticipate bad decks (Bechara et al., 1999; Bechara et al., 1998). Areas involved in executive function areas are also active (Li et al., 2010). However, lesion studies have shown mixed results for the degree of involvement of the dlPFC, which has roles in decision-making and goal-oriented behavior (Fellows and Farah, 2005; Manes et al., 2002; Bechara et al., 1998). Lastly, the supplementary motor area (SMA), which contributes to planning behavioral responses is also active during the task (Li et al., 2010; Ernst and Paulus, 2005). Therefore, performance on the IGT appears to involve a network of emotional/reward processing and executive function pathways. However, there remain inconsistent findings regarding the structural regions implicated in performance.

### 1.3 Limitations of current research

In light of the limitations of computational modelling and mixed neurophysiological findings, recent research has aimed to describe the decision-making process within the IGT more precisely. Largely, these lines of research have focused on variations in design of the task and exploring individual difference factors.

#### 1.3.1 Methodology

Although the original task has been successfully used to characterize performance across several clinical groups, one limitation of many current IGT study designs is the tendency to favor overall game performance as the sole means of analysis, which may limit a detailed exploration of the processes involved. In this regard, a modified version was developed, where the participant would pass or play on a preselected deck (Cauffman et al., 2010; Peters and Slovic, 2000). In this design, participants cannot simply avoid selecting a deck, and instead are forced to choose whether to play a presented deck or pass on it. This allows for further analysis by isolating the response to each specific deck (Peters and Slovic, 2000).While it is unclear if this version utilizes differing cognitive processes from the original IGT format, it has been suggested that this design permits for the examination of different stages of the decision-making process, particularly when using ERPs (Cui et al., 2013). Cui et al. (2013) highlight that the decision-making process requires at least three stages; evaluating the nature of each deck, deciding on a response, and then processing the consequences. Thus, in the standard IGT, a participant’s choice of a deck reflects the contribution of multiple processes that may not be clearly captured by measuring performance of feedback alone. While measuring overall game performance can provide an estimate of learning in the task, it may limit the examination of the information processing styles that may underlie that variability in behavior observed across studies.

In contrast with ERPs, measures such as fMRI and SCR may be limited in their ability to measure these stages because of their poor temporal resolution (Menon and Crottaz-Herbette, 2005; Dunn et al., 2006). Therefore, it has been argued that ERP’s may additionally provide a useful measure to explore the decision-making process in the IGT (Cui et al., 2013). In general, ERPs are derived from EEG signals, the recording of voltage on the scalp, which in turn is generated from neuronal activity (Marino and Mantini, 2024). Unlike fMRI and SCR, EEG is temporally sensitive, allowing the recording of the brain’s physiological response during each decision-making stage (Michel and Murray, 2012; Menon and Crottaz-Herbette, 2005; Dunn et al., 2006). It is also relatively cheap and non-invasive compared to other imaging techniques.

A commonly mentioned disadvantage of EEG is low spatial resolution (Marino and Mantini, 2024). As the voltage produced from neural generators disperses throughout the cortex, it becomes increasingly difficult to pinpoint the location of these neural generators (Burle et al., 2015). Moreover, several neuronal generators produce similar spatial polarities on the scalp (Marino and Mantini, 2024). However with the advent of high density EEG systems and more sophisticated head mapping and source localization algorithms, potential neural generators can be found (Marino and Mantini, 2024; Michel and Brunet, 2019). The caveat of this approach is the lack of certainty for these neural generators (Woodman, 2010); however this approach combines the benefit of using the temporal sensitivity of EEG with identifying possible sources of these cognitive processes. Overall, incorporating EEG with the modified IGT would allow for the investigation of cognitive processes in each decision making stage while further providing some insight into the active brain regions involved (Cui et al., 2013; Michel and Brunet, 2019).

#### 1.3.2 Individual difference effects on task performance

Current research has largely ignored how individual differences may adversely affect performance on the task (Chandrakumar et al., 2018). There is evidence that some control groups favor risky decks on the IGT (Bechara and Damasio, 2002; Steingroever et al., 2013; Ma et al., 2015; Lin et al., 2008). Several factors could explain this result such as sensitivity to loss frequency as mentioned above (Garon and Longard, 2015; Steingroever et al., 2013), high reward responsiveness and sensation-seeking behavior (Buelow and Suhr, 2013) as well as negative affect (Suhr and Tsanadis, 2007; Buelow and Suhr, 2013). Some studies have demonstrated that individuals who are impulsive choose more risky deck selections (Franken et al., 2008; Buelow and Suhr, 2013; Sweitzer et al., 2008), while others have found no such relationship (Upton et al., 2011). So, despite the widespread literature on clinical groups’ performance in the IGT, more work is needed to explore the contribution of individual differences affecting learning on the task (Buelow and Cayton, 2020).

For instance, symptom-based covariates appear to contribute to decision-making in clinical populations. Although, it is argued that depression affects reward processing (Admon and Pizzagalli, 2015) the performance deficits in the IGT are largely mixed. While some studies show that depressed individuals select more disadvantageous decks than controls (Must et al., 2006; Cella et al., 2010; Moniz et al., 2016), other studies have shown those with MDD select more advantageous decks (Smoski et al., 2008) or equal rates of selections compared to controls (McGovern et al., 2014; Gorlyn et al., 2013). Lack of evidence for consistent deficits may speak to the heterogeneity of the disorder, such as differences in levels of apathy, anhedonia, or information-processing styles (Must et al., 2013; McGovern et al., 2014). For example, McGovern et al. (2014) have shown that MDD participants with high levels of apathy were associated with an increased selection of advantageous decks compared to controls. It was argued that insensitivity to rewards or a lack of motivation to seek rewards led to a more conservative playing style.

As highlighted previously, computational modelling approaches have shown promise for exploring the characteristics of different populations, however more work needs to be done to determine the appropriate models as well as their parameters. Incorporating ERPs into the study design first may allow for a more detailed exploration of information processing styles, motivational factors, and how they interact throughout the decision-making process, allowing for better mechanistic clarity to inform modeling assumptions.

### 1.4 Focus of this review

Altogether there is a need to clarify the decision-making process within the IGT. As EEG can provide an in-the-moment measurement of cognitive processing, the next part of the review will highlight studies that have used ERPs in the IGT. First, a review of the studies that have used the standard IGT will be conducted. Next, studies using a modified version of the task (Cauffman et al., 2010; Peters and Slovic, 2000) that represents an information approach will be conducted. As mentioned above, this adaptation allows the characterization of each decision-making stage and the recording of the behavioral and electrophysiological response for each deck (Cauffman et al., 2010; Cui et al., 2013; Peters and Slovic, 2000). In addition, this version ensures all participants use a similar search strategy, as opposed to the original IGT where participants can select all the cards from one deck (Cauffman et al., 2010; Peters and Slovic, 2000). In this way, the modified version may allow for the determination of the underlying processes contributing to individual differences in task performance. Furthermore, this review will discuss moderators of ERP amplitudes and examine the utility of the modified IGT for understanding the nature of decision-making difficulties in clinical populations, using depression as an example.

### 1.5 Event-related potentials included in the review

This review will focus on summarizing the few studies that have used ERPs to parse out the cognitive processes in the different decision-making stages of the IGT. ERPs are neural responses specific to an event of interest (Woodman, 2010). They are derived from averaging multiple EEG signals, which isolates the cognitive processes during the event. The first few peaks of the resulting waveform are normally considered to be sensory and perceptual processes while mid-range and later stage potentials reflect more internal cognitive functions such as information processing (Sur and Sinha 2009).

There are several common ERPs used across the IGT studies: the feedback-related negativity (FRN), P300, and the Decision Preceding Negativity (DPN). First, the FRN is a negative peak between 230 to 330 ms after performance feedback (Gehring and Willoughby, 2002; Miltner et al., 1997; Badgaiyan and Posner, 1998), reflecting the early evaluation of outcomes that are worse than expected (Cui et al., 2013; Wu and Zhou, 2009). It is distributed in the medial-frontal and central regions and is thought to originate from the Anterior Cingulate Cortex(ACC), a structure involved in error processing (Gehring and Willoughby, 2002; Miltner et al., 1997; Badgaiyan and Posner, 1998). Next, the P300 is a positive component elicited between 200 ms and 500 ms after stimulus onset or feedback (Martínez-Selva et al., 2019). It is generated from orienting attention to novel stimuli (Squires et al., 1975) and updating working memory from task relevant stimuli (Conroy and Polich, 2007; Squires et al., 1975; Donchin, 1981). It can either have a frontal-central or parietal distribution depending on the context (Polich, 2007). The frontal-central distribution is thought to arise from the prefrontal cortex allocating attention to novel stimuli (Knight, 1984; Polich, 2007; Knight, 1984), while the parietal distribution is seen during the updating of working memory (Polich, 2007) and has been shown to involve the tempo-parietal junction/TPJ (Knight et al., 1989). Finally, the DPN, is a slow negative cortical wave occurring before settling on a choice, represents the anticipation of risky decisions (Bianchin and Angrilli, 2011; Dong et al., 2016).

Less frequently researched are the early negative wave, the P200, and late potentials. The early negative wave is a frontal to central negative peak appearing between 80 to 180 ms after feedback, thought to reflect the early processing of positive or negative feedback (Martínez-Selva et al., 2019). The P200 is a positive peak at frontal electrodes between 180 and 280 ms, representing the first processing distinction between good and bad outcomes. The late potentials—a positive potential in the central to parietal area after 300 ms, and negative potential that is frontally distributed between 450 and 800 ms after feedback—reflect the emotional processing of significant outcomes (Hajcak and Foti, 2020; Martínez-Selva et al., 2019). Altogether, these components reflect neural activity that are sensitive to attention, working memory and emotional factors. Thus, ERPs may provide an objective way to explore the cognitive processes relevant to decision-making. Next, we provide a review of ERP findings in the standard version of the IGT and afterward findings from the modified IGT.

## 2 Method

Google Scholar and Proquest databases were searched for relevant articles using the phrase (“Iowa Gambling” OR IGT) AND (ERP or “event related”), which was last searched on August 2nd, 2024. Studies were included if they met the following criteria: (I) examined the performance of clinical or control populations on the Standard IGT or the Modified version (Cauffman et al., 2010; Peters and Slovic, 2000) and (II) recorded EEG during the IGT and then analyzed the data collected to identify specific event-related potentials (ERPs). The authors excluded studies containing versions of the IGT with two fixed amounts visible to participants. In this gambling paradigm, only the probability of loss is varied and as such, distinguishing the more advantageous option might be easier on these modifications compared to the standard IGT. Relevant articles were identified through screening titles and abstracts, and only those with available full texts in English were selected to be included in this review. The authors extracted the following information from the articles: participant characteristics including age, gender and clinical diagnosis, sample size, ERPs observed and major findings.

## 3 Results

From our search, 15 studies were found using the standard version of the IGT while three studies used the modified version with EEG (Cauffman et al., 2010; Peters and Slovic, 2000).

### 3.1 Standard IGT

The prominent decision-making stage investigated in the standard IGT is feedback processing. Specifically, most of the research focuses on FRN and P300. Few studies have investigated ERPs in the choice evaluation and response selection stages, possibly because these stages are confounded in the standard task. This version does not separate temporally the responses associated with surveying all potential decks in the choice evaluation stage and finalizing a choice in the response selection phase (Cui et al., 2013). As a result of this limitation, this review will combine these two decision-making stages under this version.

#### 3.1.1 Choice evaluation/response selection

##### 3.1.1.1 DPN

Two studies have measured the amplitude of the DPN before participants’ responses using the standard IGT (Giustiniani et al., 2015; Bianchin and Angrilli, 2011). Disadvantageous decks evoke more negative DPN response in the right prefrontal area, inferred to result from greater attentional allocation to bad decks (Bianchin and Angrilli, 2011; Giustiniani et al., 2015). Furthermore, these decks evoke a less negative peak in the left central pre-motor area, thought to reflect the inhibition of planned motor responses for bad decks (Bianchin and Angrilli, 2011). The DPN is not related to performance as good and bad performers show similar DPN responses on the standard IGT (Giustiniani et al., 2015).

#### 3.1.2 Feedback processing

##### 3.1.2.1 FRN

The FRN is the first component depicting a consistent and marked difference between the processing of rewards and punishments (Miltner et al., 1997; Yeung and Sanfey, 2004; Holroyd et al., 2006). Correspondingly, the standard IGT has shown that FRN amplitude is generally increased in response to losses than wins (Garrido-Chaves et al., 2021; Garrido-Chaves et al., 2020; Martínez-Selva et al., 2019; Di Rosa et al., 2017; Ba et al., 2016; Balconi et al., 2015; Bianchin and Angrilli, 2011; Schuermann et al., 2011; Azcárraga-Guirola et al., 2017). However, some studies have also found the opposite effect (Zhang et al., 2024; Liu et al., 2022; Mapelli et al., 2014; Tamburin et al., 2014). It has been proposed that the FRN functions partly to reconcile discrepancies between predictions and novel outcomes, where unexpected occurrences elicit larger FRN amplitudes and signal a need to update models required for learning (Wu and Zhou, 2009; Oliveira et al., 2007; Holroyd and Coles, 2002). Indeed, individuals who displayed larger FRN magnitudes selected more advantageous choices in the IGT across trials (Schuermann et al., 2011; Martínez-Selva et al., 2019). A potential limitation of these findings is that the size of the loss might be accounting for the observed result instead of its valence. Future studies should disentangle the effects of magnitude on FRN amplitude separate from valence while using the standard IGT.

##### 3.1.2.2 P300

P300 is normally observed following the FRN and is thought to signify attention and later-stage processing such as the updating of working memory (Polich, 2007; Donchin, 1981). Similar to the FRN, the standard IGT shows a valence-type relationship for P300, where losses evoke higher P300 magnitudes than wins (Cui et al., 2013; Liu et al., 2022; Garrido-Chaves et al., 2020; Martínez-Selva et al., 2019; Balconi et al., 2015; Giustiniani et al., 2015). However, a few studies depict wins eliciting higher P300 amplitudes (Ba et al., 2016; Guo et al., 2019; Mapelli et al., 2014; Tamburin et al., 2014) or equal responses to gains and losses (Schuermann et al., 2011). Interestingly, in a modification of the task where participants choose between two cards, participants had higher responses when given nothing versus a loss (Azcárraga-Guirola et al., 2017). Heightened P300 amplitudes in response to losses might occur because of their unexpected and motivationally relevant nature (Balconi et al., 2015; Martínez-Selva et al., 2019), which in turn, would necessitate greater resource allocation for encoding (Polich, 2007). Differences in P300 amplitudes can further be used to predict performance. Giustiniani et al. (2015) showed that those who chose more advantageously distinguished between losses and wins in their P300 amplitude. This result contrasts with those of Martínez-Selva et al. (2019), who showed that P300 is unrelated to the performance on the IGT.

Since P300 is often sensitive to novelty (Squires et al., 1975) task factors such as magnitude or frequency of loss may be expected to modulate its amplitude. However, there is some evidence that losses still evoke greater P300 responses than wins when controlling for the magnitude of the outcome (Giustiniani et al., 2015). Notably, no study to date has accounted for the effects of frequency on P300 amplitude differences separate from valence.

##### 3.1.2.3 Other components

To the best of our knowledge, only a few studies have focused on early and late processing ERP components using the standard IGT. One study has highlighted the presence of the early negative wave (ENW), which is thought to reflect early stage processing of outcomes (Martínez-Selva et al., 2019). Losses evoked similar amplitudes to wins at frontal and central electrodes, but higher than wins at parietal electrodes. In addition, the amplitude of ENWs elicited from losses and wins was positively related to the number of advantageous card selections. The author suggests this evaluation might be more general as both losses and wins predict behavior.

The P200 is observed after the ENW and represents the early processing of feedback based on expectancy and valence (Polezzi et al., 2008, Schuermann et al., 2012). While some studies show that rewards and punishments elicit equal P200 magnitudes (Mapelli et al., 2014; Giustiniani et al., 2015), Martínez-Selva et al. (2019) demonstrated a higher P200 amplitude to losses than wins. Differences in how each study illustrated wins and losses might explain some discrepancies. Like the FRN, P300, and ENW, the P200 can be linked to behavioral outcomes on the IGT. Giustiniani et al. (2015) showed that people who played advantageously had higher P200 responses for both wins and losses than those who had played disadvantageously. This result contrasts with those from Martínez-Selva et al. (2019), who showed that the higher the P200 response to loss, the greater number of choices from disadvantageous decks.

Two studies have characterized late latency potentials on the standard IGT (Martínez-Selva et al., 2019; Liu et al., 2022). Late potentials index the extent of emotional reactivity to feedback (Martínez-Selva et al., 2019; for a review see Hajcak and Foti, 2020). For the late positive potential (LPP), Liu et al. (2022) found that reactions to losses produced a higher LPP than wins. Martínez-Selva et al. (2019) focused on the late negative potential (LNP), which was more negative for losses than wins at frontal and central electrodes. Furthermore, late potentials can predict behavior as LNP amplitudes from gains at parietal areas were positively associated with the number of choices from advantageous decks.

#### 3.1.3 Moderators of ERPs

##### 3.1.3.1 Individual difference factors

Four studies have examined how individual differences are associated with neural responses on the standard IGT. The factors studied include individual differences in behavior activation (Balconi et al., 2015), gender (Garrido-Chaves et al., 2020), and age (Garrido-Chaves et al., 2021; Di Rosa et al., 2017).

Behavior activation(BAS) is an individual’s tendency to experience positive emotions upon receiving rewards and readily seek them out (Carver and White, 1994). Individuals high in BAS actively approach rewards in the environment and have high drives to accomplish goals. Balconi et al. (2015) showed that the high BAS group did not distinguish between wins and losses, while the low BAS group had higher FRN responses to losses than wins. Moreover, the high BAS group had smaller P300 amplitudes for losses when compared to the low BAS group. These results suggest that those high in BAS inadequately processed feedback, leading to poor performance.

Demographic factors may also affect feedback processing. Even though both genders perform equally well on the IGT, men there is some evidence that men do not distinguish between wins and losses while women are more responsive to losses than gains in their FRN (Garrido-Chaves et al., 2020). Results are mixed regarding the effect of age on FRN and P300 amplitude (Garrido-Chaves et al., 2021; Di Rosa et al., 2017).

##### 3.1.3.2 Clinical groups

Furthermore, standard IGT results indicate consistent performance deficits and ERP modulations across a multitude of clinical populations including those with Parkinson’s, Multiple Sclerosis (MS), and Borderline Personality Disorder or BPD (Mapelli et al., 2014; Azcárraga-Guirola et al., 2017; Schuermann et al., 2011) These populations were observed to have non-significant differences in their FRN response between losses and gains during outcome evaluation. Given the FRN’s potential role in reinforcement learning, an inability to distinguish between positive prediction errors (PPE) and negative prediction errors (NPE) through the FRN could explain some of the performance deficits (Pfabigan et al., 2011). Similar findings have been observed for the P300, which were also not significantly different between losses and gains in the MS and Parkinson’s populations (Mapelli et al., 2014; Azcárraga-Guirola et al., 2017), perhaps suggesting downstream effects from the faulty initial processing of feedback. Altogether, differences in reward processing may lead to a failure in these populations to encode separate deck evaluations across the task, despite distinct punishment schedules.

### 3.2 Modified IGT

The modified IGT reviewed is the version by Peters and Slovic (2000), which allows individuals to pass or play on a highlighted deck. This version allows for the complete characterization and separation of ERPs observed in three decision-making stages: choice evaluation, response selection, and feedback processing (Cui et al., 2013). In this version the following findings have been observed.

#### 3.2.1 Choice evaluation

##### 3.2.1.1 P300

Three studies have used the modified version of the IGT to investigate P300 in the choice evaluation stage (Guo et al., 2019; Cui et al., 2013; Dong et al., 2016). Disadvantageous decks evoked larger P300 responses than advantageous decks, reflecting more attention and working memory processes given to bad decks (Guo et al., 2019; Dong et al., 2016; for a review Conroy and Polich, 2007). The modified version further allows for a more accurate investigation of how attentional processes during the choice evaluation stage could influence subsequent behavioral responses (Cui et al., 2013). The larger the choice evaluation P300, the more the participants decided to pass than play, specifically for disadvantageous decks.

#### 3.2.2 Response selection

##### 3.2.2.1 DPN

Two studies have used the modified version to assess the DPN (Cui et al., 2013; Dong et al., 2016). Because of its design, participants either pass or play on a single deck, allowing the characterization of the specific behavioral and neural response for each deck (Cauffman et al., 2010; Peters and Slovic, 2000). In the modified version, the DPN is thought to represent the anticipation associated with making a decision (Cui et al., 2013; Dong et al., 2016). Higher DPNs were associated with passes than for plays, specifically when it came to passing on disadvantageous decks (Cui et al., 2013). This outcome might result from the DPN influencing subsequent behavior in keeping with the somatic marker hypothesis (Cui et al., 2013; Dong et al., 2016; Bechara and Damasio, 2005).

#### 3.2.3 Feedback processing

##### 3.2.3.1 FRN and P300

In relation to feedback processing, the modified version has shown similar results to those of the standard IGT. Losses evoke greater FRN and P300 amplitudes than wins (Dong et al., 2016; Cui et al., 2013). This finding suggests that losses elicit more later-stage processing of feedback than wins. Furthermore, P300 could also serve to provide additional information not yet accounted for by the FRN in the initial valence classification. Larger losses elicit greater P300 responses than smaller losses, indicating that P300 might allow the updating of models based on the size of the outcome (Cui et al., 2013). More studies should incorporate and test whether this observed result is consistent across studies.

The observed difference between wins and losses further cannot be attributed to the fact that losses occur less frequently than wins on the IGT. By comparing decks with similar loss frequency, Cui et al. (2013) found that losses still elicit greater FRN and P300 amplitudes than wins, suggesting that the intrinsic nature of losses contributes to this increased processing instead of frequency.

##### 3.2.3.2 Other components

To our knowledge, no study has used the modified version to investigate early and late-stage feedback processing on the IGT. Similar to the standard version, the ENW, P200, and late potentials may be expected to reflect individual differences in performance.

#### 3.2.4 Moderators

##### 3.2.4.1 Individual differences

To date, one study has used the modified version to examine the effect of individual differences on IGT performance. Dong et al. (2016) showed that cognitive flexibility predicted performance on the task. Cognitive flexibility was determined by the WCST, a standard measure of task switching and implicit learning (Gamboz et al., 2009; Dong et al., 2016). Those low in cognitive flexibility had similar P300 amplitudes when observing good and bad decks, suggesting an inability to distinguish between decks (Dong et al., 2016). Furthermore, they showed opposite DPN responses to the cognitive flexible group when selecting a response in the latter trials of the IGT. Those low in cognitive flexibility showed greater negativity towards playing than passing. This result might suggest a delay in learning in those low in cognitive flexibility. When responding to feedback, those low in cognitive flexibility had higher FRN responses for wins than losses while the cognitive flexible group had the opposite effect. The authors show that a higher FRN response in this group was related to a smaller P300 amplitude in the choice evaluation stage, suggesting that their abnormal processing of wins was related to an inability to distinguish between good and bad decks.

##### 3.2.4.2 Clinical groups

At the time of writing this review, no study has used the modified version to explore decision making in clinical groups.

### 3.3 Summary modified vs. standard IGT

The 15 studies included in this review have explored ERPs associated with the standard version of the IGT while three studies have used the modified version (Cauffman et al., 2010; Peters and Slovic, 2000). Most standard versions of the task have solely focused on the ERPs evoked by the feedback stage, specifically the FRN and P300. These studies have shown that losses evoke higher FRN and P300 amplitudes, indicating a greater need to process information from losses than wins (Liu et al., 2022; Garrido-Chaves et al., 2021; Garrido-Chaves et al., 2020; Martínez-Selva et al., 2019; Di Rosa et al., 2017; Ba et al., 2016; Balconi et al., 2015; Giustiniani et al., 2015; Bianchin and Angrilli, 2011; Schuermann et al., 2011; Azcárraga-Guirola et al., 2017). In addition, evidence supports the idea that FRN and P300 amplitudes are related to behavioral outcomes on the IGT (Schuermann et al., 2011; Martínez-Selva et al., 2019; Giustiniani et al., 2015). On the other hand, ERPs occurring in early—and late-stage processing such as the ENW, P200, and late potentials are largely understudied (Liu et al., 2022; Martínez-Selva et al., 2019; Mapelli et al., 2014; Giustiniani et al., 2015). These ERP components are key facets in uncovering the full extent of perceptual and emotional processing in decision-making (Bourisly and Shuaib, 2018; Hajcak and Foti, 2020), particularly in situations where the decision-making process goes awry such as in clinical populations. Research on the standard IGT incorporating ERPs has illustrated that several clinical populations such as those with Parkinson’s, MS, and BPD fail to distinguish between losses and wins in their FRN and at times in their P300 amplitudes (Mapelli et al., 2014; Azcárraga-Guirola et al., 2017; Schuermann et al., 2011). Studying late-stage potentials would highlight how emotional dysregulation could interfere with the decision-making process in clinical populations.

Less studied is the modified version (Cauffman et al., 2010; Peters and Slovic, 2000), which allows an individual to pass or play on a highlighted deck. This version allows for the characterization of ERPs in each decision-making stage and the isolation of behavioral and neural responses for each deck (Cui et al., 2013; Cauffman et al., 2010; Peters and Slovic, 2000). Consistent with the standard IGT, these studies have shown that losses elicit higher FRN and P300 amplitudes than wins, indicating more processing of losses (Dong et al., 2016; Cui et al., 2013). In addition, research using this version has characterized the P300 in the choice evaluation stage and the DPN in the response selection stage, allowing the role of attentional and anticipatory processes, respectively, to be studied on the IGT (Guo et al., 2019; Cui et al., 2013; Dong et al., 2016). However, more work is needed on the individual factors and clinical groups that moderate the decision making process in the modified version.

## 4 Discussion

The standard IGT remains a clinically relevant tool for exploring the decision-making process. Evidence suggests that ERPs elicited from feedback are associated with task performance (Schuermann et al., 2011; Martínez-Selva et al., 2019; Giustiniani et al., 2015). Larger FRN amplitudes and therefore greater early stage processing of feedback was linked to more successful outcomes on the IGT (Schuermann et al., 2011; Martínez-Selva et al., 2019). Additionally, greater P300 amplitude differences between losses and wins have been associated with better performance on the IGT (Giustiniani et al., 2015), suggesting that that level of attention allocation may play a role in learning in the task. As a result, ERPs have provided some evidence linking online neural processes to feedback response, learning and decision-making on the IGT. However, few studies have sought to clarify the role of sensory/perceptual and emotional processing as evidenced by a lack of focus on early and late components (Liu et al., 2022; Martínez-Selva et al., 2019; Mapelli et al., 2014; Giustiniani et al., 2015). A greater focus on emotion processing may also help to explore the nature of decision-making deficits in mood disorders. Furthermore, if emotion plays a prominent role in the decision-making process, it may be expected to influence performance and corresponding ERP components on the IGT across clinical populations and non-clinical populations (c.f. Bechara et al., 2000a).

The current review highlights the potential for implementing an information processing approach to explore the processes involved during learning. Using the modified IGT, recent research has isolated ERP components for choice evaluation, response selection in addition to feedback processing within the task (Cui et al., 2013). As a consequence, the effect of reduced attention to specific stimuli as observed by the choice evaluation P300 amplitudes and the role of anticipation in building associations to good and bad decks as observed in the DPN can be investigated (Dong et al., 2016). This approach may help meaningfully extend decision-making research using the IGT, particularly in the exploration of individual factors and covariates that have been argued to impact performance (Chandrakumar et al., 2018). However, to our knowledge, only one study has utilized this novel modified approach, showing cognitive flexibility to be a key process predicting overall task performance and moderating specific neural correlates occurring across each stage (Dong et al., 2016). Therefore, more research is needed to integrate the information processing approach developed by Peters and Slovic (2000) and extended by Cui et al. (2013) using ERPs.

To demonstrate how individual differences may be understood using an information processing approach in the IGT, we next describe a potential application using depression research as an example. Depression exhibits some of the key issues that may arise from third factors and methodology often described in the literature. As highlighted previously, the effect of depression on performance in the IGT has been mixed. This effort is likely constrained by heterogeneity in symptoms and their effects on reward processing, which may result in subtle differences in decision-making that are hard to capture in behavioral versions of the standard IGT in particular. For example, people characterized by apathetic depression performed better than the control group possibly because their reward insensitivity contributed to a focus on long term gain instead of immediate reward (McGovern et al., 2014). Meanwhile those who suffered from anhedonia were observed to have worse performance than the control group on another reward learning task (Vrieze et al., 2013). This difficulty in reward learning is thought to arise from an inability to update reward learning contingencies on the IGT (Must et al., 2013). Therefore, exploring the manner that perseverative behavior influences performance and moderates ERP amplitudes may hep to clarify the effect of depression on learning in the task.

For instance, individual differences in depression severity and risk is frequently associated with an increased tendency towards rumination. Rumination is defined as a repetitive, and often uncontrolled response style that is characterized by a narrowed attentional focus on the negative consequences of their depressive symptoms (Whitmer and Gotlib, 2013; Davis and Nolen-Hoeksema, 2000). Although the nature of the process of rumination is still being studied, it affects core components of decision-making, with high levels of rumination associated with attention and updating deficits (Whitmer and Gotlib, 2013), as well as cognitive inflexibility as measured by the WCST (Davis and Nolen-Hoeksema, 2000). Consistent with the information processing view of IGT performance, it is argued that rumination’s impact on cognition plays a key role in the development of poor learning and reward processing in depression (Rutherford et al., 2023). Thus, it would be expected that rumination would adversely affect the underlying mechanisms of decision-making and how choice evaluation, response selection and feedback processing are applied in the task. As highlighted in the review, measuring these processing through ERPs affords an opportunity to explore how the application of these processes unfold in real-time. Additionally, it is through rumination’s effects on learning and reward that additional risk factors such as anhedonia are thought to arise (Rutherford et al., 2023). Therefore, how rumination impacts later components associated with emotion processing, such as the LPP, may also be important to measure. If rumination is differentially associated with biased attention to negative compared positive feedback, it may then interact with the evaluation of each deck and anticipatory processing during response selection across early and into late phases of the IGT where learning is often argued to occur (cf. Bechara et al., 2000a). Overall, these examples highlight how the modified IGT has the potential to examine key factors related to information processing in decision making and clarify mixed findings in depression research and clinical research more broadly.

### 4.1 Additional avenues for future research

The modified IGT has shown promise to provide a more detailed exploration of decision-making within the task. However, it is unknown to what degree the standard and modified IGT utilize similar mechanisms. As reviewed previously, similar ERP components are elicited across both versions of the tasks. In addition, there is evidence that behavioral performance on the modified IGT is similar to that of the standard version. In the modified IGT, high conceptual knowledge has been associated with increased net-scores across blocks compared to participants with low conceptual knowledge (Dong et al., 2016). These findings suggest that there may be significant overlap in mechanisms, however, more research is needed to clarify the potential cognitive impact of experimentally controlling deck selection opposed to free choice. Beyond this, future research will be needed to determine how well other measures, such as switching rates and rate of selection of decks differing in loss frequency, are similar across tasks. Furthermore, the review highlighted the benefits and potential limitations of computational modeling IGT data to identify individual differences in performance. Future research that integrates ERP components within model comparisons may offer a way to help generalize models and facilitate model selection beyond fit indices. This reflects a computational cognitive neuroscience approach, which has been argued to benefit the computational field by ensuring selected models accurately reflect neurobiological function (Ashby and Helie, 2011; Hawkins et al., 2024).

## 5 Conclusion

The IGT is a sophisticated and sensitive task design that has provided consistent research over two decades demonstrating its utility in measuring risk-taking in both clinical and typical populations. Through variation in design and analysis technique, the nature of learning in the task, and its link to underlying neural and physiological mechanisms can be made. Thus, the research using the IGT seems aligned to build on findings of the past and contribute to a greater understanding of the nature of information processing in decision-making.
